# The association between the knowledge, perception, and practice of dietary supplement among Chinese adults

**DOI:** 10.3389/fnut.2024.1493504

**Published:** 2024-11-08

**Authors:** Din Son Tan, Xijie Wang, Xuechen Zhao, Ai Zhao

**Affiliations:** ^1^Vanke School of Public Health, Tsinghua University, Beijing, China; ^2^Institute for Healthy China, Tsinghua University, Beijing, China; ^3^Regenerative Bio Cooperation, Hangzhou, China

**Keywords:** dietary supplement, Chinese adults, a nationwide cross-sectional study, nutrition knowledge, behavior model

## Abstract

**Introduction:**

Rapid growth has been found in the market of dietary supplements (DSs) in China. However, studies about the knowledge level, intentions, and behavior related to DS remained limited in the Chinese population. This study aimed to explore the knowledge level, perception, and practice toward DS among Chinese adults.

**Methods:**

This is a cross-sectional design among 1,714 participants aged between 18 and 65 years. A total of 54.1% of participants reported purchasing supplements in the past 6 months. Knowledge levels were assessed with a score from 1 to 10 based on correct answers to 10 binary questions about supplements.

**Results:**

Only 29.1% of participants answered seven or more questions correctly, while 7.9% answered three or fewer questions correctly. Participants with high and middle knowledge levels were more likely to seek medical advice when experiencing discomfort symptoms and were less likely to choose DS, with corresponding odd ratios (ORs) of 1.58 (95% CI: 1.16, 2.13) and 0.69 (95% CI: 0.52, 0.91) in high knowledge group and ORs of 1.36 (95% CI: 1.03, 1.80) and 0.96 (95% CI: 0.72, 1.30) in middle knowledge group (*P*_for trend_ = 0.003 and 0.028, respectively). No significant differences were found in the motivation of DS use between knowledge levels. Although higher knowledge levels were associated with less spending on DS (OR _high_ = 0.69; 95% CI: 0.49, 0.99; OR _middle_ = 0.88; 95% CI: 0.64, 1.20; *P*_for trend_ = 0.038), it did not guarantee better and more accurate awareness toward DS use.

**Discussion:**

In conclusion, there is a growing demand for supplements among Chinese adults, but a significant gap between knowledge and behavior exists, affecting decision-making behaviors regarding DS.

## Introduction

According to the World Health Organization (WHO), non-communicable diseases (NCDs) such as cardiovascular diseases, cancer, diabetes, and chronic respiratory diseases are the leading cause of death globally, accounting for 74% of total deaths worldwide (1), and the trend is expected to continue rising (2). There is a need to shift the focus from traditional “seeking medical treatment” to a proactive approach centered around prevention and promoting a healthy lifestyle (3, 4). As the social economy continues to develop, there is a noticeable increase in the consumption of healthcare products (5, 6). It has been observed that inappropriate dietary habit is associated with significantly lower diet quality among both children and adults (7). Dietary supplements (DSs), on the other hand, can help supplement one’s diet and meet basic nutritional needs (8). Although DS may not be promoted for the prevention or treatment of illness, it is common for many consumers to use them to ensure proper nutrition, reduce the risk of age-related disorders, and preserve health (9).

Legally, DSs are categorized as food and should not make therapeutic claims. However, it is common for consumers to use DSs for the prevention or treatment of illnesses, despite the lack of evidence supporting such use (10–13). Adverse events associated with supplements can be challenging to identify, and products are often withdrawn from the market only after a significant number of adverse events have occurred (14). The limited evaluation of efficacy can be attributed, at least in part, to the inherent challenges associated with assessing the effectiveness of these supplements (15). Therefore, it is important for consumers to understand the limitations of DS applications when considering the use of such products (16).

Research conducted across multiple countries has revealed widespread confusion among different groups regarding the concept of supplements (16). A nationwide cross-sectional anonymous questionnaire survey of 30,635 participants in China reported that among the surveyed residents aged 18 to 55 years, only 23.0% were able to correctly identify the main purpose of DS as compensating for dietary inadequacies (17). Another study conducted in Wuhan, China, also indicates that the overall awareness rate of nutrition and health knowledge among its residents is not optimistic (18). Better knowledge level, on the other hand, also does not necessarily lead to correct behavior. In Japan, where supplements are popular and where there is a Health Food Network consumer navigation site to provide all official information about supplements freely for the public, people were found to have extremely low awareness of appropriate use, with awareness of such information sources below 5% (19, 20). Even with guidance and advice from health professionals, people used supplements and medicines concomitantly, with an inappropriate perception of their safety and efficacy (21). Such behavior may cause serious health damage, especially to people with specific health conditions (22).

However, evidence on the theoretically based behavioral patterns of Chinese populations regarding DS use and the related influencing factors remains scarce. Previous studies in China have either been limited to a single location and a narrow age range or not focused on dietary supplements, thereby lacking a theoretical basis for their analyses and providing only statistical correlations. Therefore, the aim of this study, utilizing a nationwide cross-sectional study design based on two behavioral models, was to explore the knowledge level, perception, and practice toward DS among Chinese adults and the potential influencing factors. We hypothesize that the overall knowledge level regarding DS use in Chinese adults is related to sociodemographic characteristics and could be associated with unwise DS use behaviors.

## Materials and methods

### Study population

Participants of the present study were Chinese adults aged between 18 and 65 years and were recruited from online anonymous resources. To ensure a reliable and representative sample for the Internet survey, a professional opinion pollster from different regions of China was employed. The sample size was calculated based on the cross-sectional design [(Z_1-*α*/2_/*δ*)^2^*p*(1-*p*)], where α = 0.05 and δ = 0.05. As there are no representative data on supplement-related knowledge level, *p* was set as 50% to reach the maximum statistical effectiveness. The calculated sample size was 385. To ensure the representative data from various regions (north, central, and south China), the final sample size was set to at least 1,155 participants. Participant recruitment was applied with an online questionnaire survey platform named “Pollster” with a convenience sampling procedure. The final number of included participants was 1,714. The survey was completely anonymous and has been reviewed by the Ethics Committee of Tsinghua University (THU01-20240037).

### Questionnaire survey

A combination of the health action process approach (HAPA) and Attention, Interest, Desire, Action (AIDA) models was used to provide a theoretical framework (23, 24). The hypothetical framework of our study is displayed in Supplementary Figure S1. The questionnaire contained six parts, namely, sociodemographic characteristics, DS-related knowledge, behavior intention, attention and action, dietary quality, and health self-efficacy.

Sociodemographic characteristics included year of birth, educational background, exercise habits, occupation (medical-related or not), and family income. DS-related knowledge was evaluated with 10 binary questions based on previous studies and further developed by our research team (17, 25) (Supplementary Table S1). Cronbach’s alpha coefficient of the 10 questions was 0.6349. In factor analysis, most of the items had a uniqueness of above 0.75, while two items had a uniqueness between 0.55 and 0.6. However, as these two items (items 2 and 3) included important misconceptions about DS use, they were retained in the analyses. DS-related behavior intention, attention, and actions included the motivation, purchase behavior of DS products, and average expenses on DS products. Dietary quality was evaluated with the Dietary Diverse Score (DDS), which was based on the experience of whether 10 food groups were consumed on the day prior to the investigation date (26, 27). The participant got one point if he or she consumed something at least one time from a unique food group in 24-h dietary records. A higher score indicated better dietary diversity. Self-efficacy was evaluated with the General Self-efficacy Scale, which contains 10 items along with a 4-point Likert-type scale for each item (28). The total score ranged between 1 and 4, with a higher score indicating better self-efficacy. Rigid quality control was conducted during data collection. Duplicated IPs (*n* = 32), responses answered less than 120 s (*n* = 7), and responses with wrongly answered quality control questions (*n* = 0) were removed from the final dataset.

### Statistical analysis

Sociodemographic characteristics of participants, including their gender, year of birth (1960s or before, 1970s, 1980s, 1990s, 2000s, and after), educational background, exercise habit, occupation (medical-related or not), and family income, were all categorical variables and summarized into numbers and frequencies. The results of DDS were transformed into scores from 1 to 10. The distribution of health self-efficacy scores ranged between 1 and 4 and had a cutoff at 2.5. Participants were therefore categorized as having low self-efficacy and high self-efficacy. The chi-square tests were applied to analyze the differences in distribution of knowledge, intention, and behavior among sociodemographic groups. The associations between DS-related knowledge level, intention, and behaviors were analyzed with ordered logistic regression models or multinomial logistic regression models. Generalized structural equation modeling based on logit or ordinal logit models was applied to build the pathways between sociodemographic characteristics, DS-related knowledge, intention, and behaviors. Variables for the pathway analysis were included based on their statistical significance in preliminary analyses and their theoretical relevance to the behavioral models. Health self-efficacy and DDS were adjusted when applicable. A *p*-value of <0.05 was considered statistically significant. All analyses were performed with Stata 14.0 software (College Station, TX, United States).

## Results

### Characteristics of the participants

Among the total of 1,714 participants, 54.1% (N = 928) of them reported that they had bought supplements during the past 6 months (Table 1); 46.8% of participants who bought supplements were born in the 1980s or before and were significantly older than those who did not buy DS (35.3%; χ^2^ = 50.723, *p* < 0.001). More than one-third (36.8%) of participants who bought supplements reported a yearly income of ≥300,000 RMB, and the proportion was significantly higher than those who did not buy DS (25.5%, χ^2^ = 65.963, *p* < 0.001).

**Table 1:** Demographic characteristics of participants by DS purchase behavior over the past 6 months.

Demographic characteristics	Total *N* (%)	DS purchase		*χ*^2^/*t*	*p*- value
		Yes	No		
Sample size	1714	928 (54.1)	786 (45.9)		
Geographic area					
North	698 (40.7)	370 (53.0)	328 (47.0)	1.142	0.565
Middle	613 (35.8)	331 (54.0)	282 (46.0)		
South	227 (24.5)	227 (56.3)	176 (43.7)		
Birth date				50.723	<0.001
1960s	122 (7.1)	81 (8.7)	41 (5.2)		
1970s	230 (13.4)	138 (14.9)	92 (11.7)		
1980s	360 (21.0)	215 (23.2)	145 (18.4)		
1990s	472 (27.5)	273 (29.4)	199 (25.3)		
2000s	530 (30.9)	221 (23.8)	309 (39.3)		
Educational background				3.891	0.421
High school or below	176 (10.3)	88 (9.5)	88 (11.2)		
Associate degree	560 (32.7)	297 (32)	263 (33.5)		
Bachelor’s degree	863 (50.4)	475 (51.2)	388 (49.4)		
Master’s degree	84 (4.9)	52 (5.6)	32 (4.1)		
Doctor’s degree	31 (1.8)	16 (1.7)	15 (1.9)		
Occupation				4.247	0.120
Non-medical-related	1,185 (69.1)	624 (67.2)	561 (71.4)		
Medical marketing	348 (20.3)	195 (21)	153 (19.5)		
Medical research/teaching	181 (10.6)	109 (11.7)	72 (9.2)		
Yearly income (k)				65.963	<0.001
<100 k	622 (36.3)	258 (27.8)	364 (46.3)		
100-200 k	381 (22.2)	222 (23.9)	159 (20.2)		
>200-300 k	170 (9.9)	107 (11.5)	63 (8.0)		
>300-400 k	103 (6.0)	67 (7.2)	36 (4.6)		
>400-500 k	99 (5.8)	59 (6.4)	40 (5.1)		
>500 k	339 (19.8)	215 (23.2)	124 (15.8)		
Exercise habit				3.256	0.196
No	487 (28.4)	253 (27.3)	234 (29.8)		
Occasionally	540 (31.5)	285 (30.7)	255 (32.4)		
Yes	687 (40.1)	390 (42)	297 (37.8)		
Health self-efficacy				1.349	0.245
Low	371 (21.7)	191 (20.6)	180 (22.9)		
High	1,343 (78.4)	737 (79.4)	606 (77.1)		
DDS (mean and SD)	5.0 (1.7)	5.0 (1.7)	5.0 (1.7)	0.264	0.792

DS, dietary supplement; DDS, dietary diversity score.

A total score of 1 to 10 was calculated for the DS-related knowledge level of each participant; 29.1% of participants answered ≥7 questions correctly (categorized as high knowledge level), whereas 20.7% of them answered ≤4 questions correctly (low knowledge level). The knowledge level did not differ by age, educational background, occupation, income level, and self-efficacy (Table 2; Supplementary Figure S2). Among the 10 questions, the two questions with the lowest accuracy rates were “*All probiotics are beneficial for gut microbiota health*” and “*Health supplements can be substitute for medication*,” with accuracy rates of 39.0 and 49.3%, respectively.

**Table 2:** Knowledge level related to dietary supplements among study participants with different demographic characteristics.

Demographic characteristics	Knowledge level			*χ*^2^/*t*	*p*- value
	Low	Middle	High		
Sample size	355 (20.7)	861 (50.2)	498 (29.1)		
Geographic area				8.763	0.067
North	123 (17.6)	353 (50.6)	222 (31.8)		
Middle	139 (22.7)	304 (49.6)	170 (27.7)		
South	93 (23.1)	204 (50.6)	106 (26.3)		
Birth date				2.207	0.974
1960s	21 (17.2)	66 (54.1)	35 (28.7)		
1970s	46 (20.0)	117 (50.9)	67 (29.1)		
1980s	75 (20.8)	184 (51.1)	101 (28.1)		
1990s	97 (20.6)	238 (50.4)	137 (29.0)		
2000s	116 (21.9)	256 (48.3)	158 (29.8)		
Educational background				8.174	0.226
High school or below	37 (21.0)	90 (51.1)	49 (27.8)		
Associate degree	115 (21.5)	289 (51.6)	156 (27.9)		
Bachelor’s degree	190 (22.0)	418 (48.4)	255 (29.6)		
Master’s degree and above	13 (11.3)	64 (55.7)	38 (33.0)		
Occupation				1.569	0.814
Non-medical-related	236 (19.9)	603 (50.9)	346 (29.2)		
Medical marketing	78 (22.4)	169 (48.6)	101 (29.0)		
Medical research/teaching	41 (22.7)	89 (49.2)	51 (28.2)		
Yearly income (k)				8.791	0.552
<100 k	123 (19.8)	309 (49.7)	190 (30.6)		
100-200 k	74 (19.4)	196 (51.4)	111 (29.1)		
>200-300 k	40 (23.5)	77 (45.3)	53 (31.2)		
>300-400 k	19 (18.5)	55 (53.4)	29 (28.2)		
>400-500 k	22 (22.2)	44 (44.4)	33 (33.3)		
>500 k	77 (22.7)	180 (53.1)	82 (24.2)		
Exercise habit				12.225	0.016
No	104 (21.4)	261 (53.6)	122 (25.1)		
Occasionally	103 (19.1)	251 (26.5)	186 (34.4)		
Yes	148 (21.5)	349 (50.8)	190 (27.7)		
Health self-efficacy				0.257	0.880
Low	74 (20.0)	186 (50.1)	111 (29.9)		
High	281 (20.9)	675 (50.3)	387 (28.8)		

### Associations between sociodemographic characteristics, knowledge, and intention

**Figure 1:**
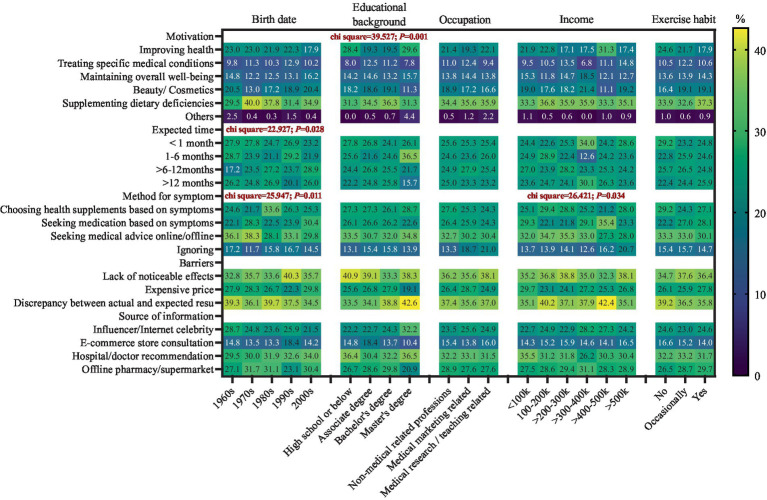
Distribution (%) of behavior intention, attention, and actions regarding dietary supplement consumption by sociodemographic characteristics.

The main reason for using a supplement was supplement for nutrient deficiency (*N* = 597; 34.8%), followed by improving health (*N* = 360; 21.0%) and for beauty reasons (*N* = 314; 18.3%); 11.1% (*N* = 190) of participants reported the main motivation for treating specific medical conditions. The proportions were 12.5 and 11.2% in participants below the undergraduate level and those with a bachelor’s degree, compared to the proportion of 7.8% in participants with a master’s degree or above (*χ*^2^ = 39.527; *p* < 0.001); 52.9% of participants would seek medication or health supplements when there are discomfort symptoms, while half of them would choose supplements rather than medications (26.8% versus 26.1% of all participants). The largest proportion of choosing health supplements were found in participants born in the 1980s (33.6%; *χ*^2^ = 25.947; *p* = 0.011), people with a yearly income of 100–300 k (29.4 and 28.8%), and people with a yearly income of >500 k (28.0%) (Figure 1). Compared to those with low DS-related knowledge levels, participants with high (OR = 1.58; 95% CI: 1.16, 2.13; *p* = 0.003) and middle (OR = 1.36; 95% CI: 1.03, 1.80; *p* = 0.031) supplement-related knowledge levels tend to seek medical advice when they have discomfort symptoms (*P*_trend_ = 0.003) and were less likely to choose health supplements (OR_high_ = 0.69; 95% CI: 0.52, 0.91; OR_middle_ = 0.96; 95% CI: 0.72, 1.30; *P*_trend_ = 0.028). There was no significant difference between medical- and non-medical-related practitioners in their choice of DS in the case of physical discomfort. However, medical practitioners were more likely to ignore symptoms of discomfort, with ORs ranging from 1.49 (95% CI: 1.09, 2.05) for medical marketing professionals to 1.73 (95% CI: 1.16, 2.56) for medical research professionals (Table 3). The results remained consistent after adjustment of sociodemographic characteristics, self-efficacy, and DDS. However, the regression analyses did not show a significant difference in motivation to choose DS in participants with different sociodemographic characteristics and knowledge levels (Supplementary Table S2).

**Table 3:** Association between sociodemographic characteristics, knowledge, and primary choice when there are discomfort symptoms.

Socio and demographic characteristics	Choose health supplements based on symptoms	Seek medication based on symptoms	Seek medical advice online/offline	Ignore
Birth date				
1960s	1 (Reference)	1 (Reference)	1 (Reference)	1 (Reference)
1970s	0.85 (0.51, 1.43)	1.39 (0.83, 2.32)	1.1 (0.7, 1.73)	0.64 (0.34, 1.19)
1980s	1.55 (0.97, 2.48)	1.02 (0.62, 1.67)	0.69 (0.45, 1.07)	0.9 (0.52, 1.57)
1990s	1.09 (0.69, 1.73)	1.11 (0.69, 1.78)	0.88 (0.58, 1.33)	0.97 (0.57, 1.64)
2000s	1.04 (0.66, 1.64)	1.54 (0.96, 2.45)	0.75 (0.5, 1.14)	0.82 (0.48, 1.39)
Educational background				
High school or below	1 (Reference)	1 (Reference)	1 (Reference)	1 (Reference)
Associate degree	1 (0.69, 1.47)	1.02 (0.7, 1.51)	0.88 (0.61, 1.26)	1.21 (0.74, 1.98)
Bachelor’s degree	0.94 (0.65, 1.35)	1 (0.69, 1.45)	0.93 (0.66, 1.32)	1.24 (0.77, 2)
Master’s degree and above	1.07 (0.64, 1.81)	0.83 (0.48, 1.43)	1.06 (0.64, 1.74)	1.08 (0.54, 2.14)
Occupation				
Non-medical-related professions	1 (Reference)	1 (Reference)	1 (Reference)	1 (Reference)
Medical marketing related	0.89 (0.68, 1.17)	0.97 (0.74, 1.28)	0.89 (0.69, 1.15)	1.49 (1.09, 2.05)
Medical research/teaching related	0.84 (0.59, 1.21)	0.89 (0.62, 1.29)	0.9 (0.64, 1.26)	1.73 (1.16, 2.56)
Yearly income				
<100 k	1 (Reference)	1 (Reference)	1 (Reference)	1 (Reference)
100-200 k	1.24 (0.93, 1.65)	0.68 (0.51, 0.92)	1.13 (0.86, 1.48)	1.02 (0.71, 1.48)
>200-300 k	1.21 (0.83, 1.77)	0.67 (0.45, 1.01)	1.16 (0.81, 1.66)	1.04 (0.64, 1.69)
>300-400 k	1.01 (0.62, 1.63)	0.99 (0.63, 1.57)	1.05 (0.67, 1.63)	0.91 (0.49, 1.7)
>400-500 k	0.8 (0.48, 1.35)	1.32 (0.85, 2.07)	0.8 (0.5, 1.28)	1.22 (0.68, 2.18)
>500 k	1.16 (0.86, 1.57)	0.73 (0.54, 1)	0.83 (0.62, 1.11)	1.64 (1.16, 2.33)
Family members				
1	1 (Reference)	1 (Reference)	1 (Reference)	1 (Reference)
2 ~ 3	0.87 (0.64, 1.19)	0.97 (0.71, 1.33)	1.03 (0.77, 1.39)	1.24 (0.82, 1.88)
4 ~ 5	0.83 (0.58, 1.2)	0.97 (0.67, 1.41)	0.97 (0.68, 1.38)	1.48 (0.92, 2.37)
≥5	0.92 (0.6, 1.42)	1.12 (0.73, 1.74)	0.81 (0.52, 1.24)	1.36 (0.78, 2.38)
Exercise habit				
No	1 (Reference)	1 (Reference)	1 (Reference)	1 (Reference)
Occasionally	0.78 (0.59, 1.03)	1.3 (0.98, 1.73)	0.99 (0.76, 1.28)	1.03 (0.73, 1.44)
Yes	0.9 (0.7, 1.17)	1.37 (1.05, 1.8)	0.87 (0.67, 1.11)	0.95 (0.68, 1.31)
Supplement knowledge level				
Low	1 (Reference)	1 (Reference)	1 (Reference)	1 (Reference)
Middle	1.09 (0.83, 1.44)	0.96 (0.72, 1.30)	1.36 (1.03, 1.80)	0.9 (0.64, 1.25)
High	0.73 (0.53, 1.00)	0.69 (0.52, 0.91)	1.58 (1.16, 2.13)	0.78 (0.53, 1.13)

After adjustment of sociodemographic characteristics, self-efficacy, and DDS score, higher supplement-related knowledge levels were associated with less expense on the supplement, with ORs of 0.88 (95% CI: 0.64, 1.20) in middle knowledge groups and 0.69 (95% CI: 0.49, 0.99) in high knowledge group (*p* for trend = 0.038; Table 4). Compared to participants with the motivation of improving overall health, participants who aimed to treat specific medical conditions (OR = 1.78; 95% CI: 1.12, 2.81; *p* = 0.014) and for beauty/cosmetic (OR = 1.56; 95% CI: 1.04, 2.33; *p* = 0.031) purposes had greater odds of higher expense on the supplement (Supplementary Figure S3).

**Table 4:** Association between supplement knowledge level, supplement purchase behavior during the past 6 months, and average expenses on supplement.

Supplement-related behavior	Supplement-related knowledge level			*p* for trend
	Low	Middle	High	
Crude model				
Purchase behavior	1 (Reference)	0.82 (0.64, 1.05)	0.76 (0.58, 1.00)	0.058
Expenses on supplement	1 (Reference)	0.91 (0.67, 1.23)	0.71 (0.51, 1.00)	0.046
Adjusted model				
Purchase behavior	1 (Reference)	0.82 (0.63, 1.06)	0.77 (0.58, 1.03)	0.090
Expenses on supplement	1 (Reference)	0.88 (0.64, 1.20)	0.69 (0.49, 0.99)	0.038

Expense on supplement was only calculated in participants who bought supplement during the past 6 months (*N* = 924). Adjusted model included variables related to the individual’s year of birth, educational background, occupation, yearly income, exercise habit, self-efficacy (low or high), dietary diversity score, and the geographic area they lived in.

**Figure 2:**
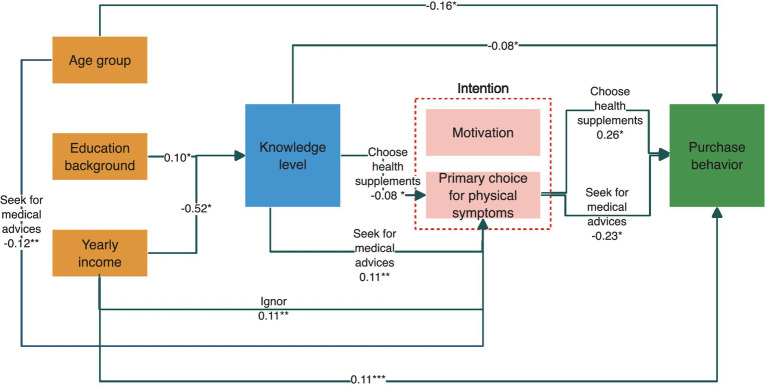
Results of generalized structural equation modeling on the relationship between sociodemographic characteristics, knowledge, intention, and behavior.

A pathway analysis was further conducted. As displayed in Figure 2, higher educational background and lower yearly income were both associated with higher knowledge levels. Participants with higher knowledge levels tend to seek medical advice when they had physical symptoms (OR = 0.11; 95% CI: 0.04, 0.18; *p* = 0.002) and were less likely to choose supplements (OR = -0.08; 95% CI: −0.15, −0.01; *p* = 0.032). Meanwhile, there was no significant association indicating pathways from higher knowledge level to DS-related motivation and purchase behavior.

## Discussion

In the present study, we developed a questionnaire based on the HAPA and AIDA models to assess the knowledge, intention, and behaviors of Chinese adults regarding dietary supplements. Using cross-sectional survey data from 1714 participants, we found that there was an increasing demand for DS among Chinese adults, while the DS-related knowledge level remained limited. Although the knowledge level was associated with DS-related intentions, it would not necessarily lead to sensible behavior toward DS.

The use of supplements has risen precipitously during the past decade, with the greatest growth found in Asia Pacific (29). During the 2010–2012 wave of China Nutrition and Health Surveillance, only less than 1% of participants reported using supplements during the past months, with the highest proportion found in participants aged 60 years and above at 1.75% (30). However, in the present study, more than half of the participants reported having bought supplements during the past 6 months, while more than half of them reported spending more than 1,000 RMB on supplements every month. The elder groups, particularly middle-aged and elderly individuals, constitute the main consumer base for supplement products (31), which matches the result in this study where 46.8% of participants who bought supplements were born in the 1980s or before. With a greater pursuit of health, they are also inclined to spend more money on purchasing supplements. However, due to limited access to information resources and a reduced ability to discern the authenticity of information, they are also more susceptible to falling victim to false advertising and misleading claims (32, 33).

Mismatched with the continuously growing demand for DS, research aimed at investigating relevant knowledge levels in Chinese residents remained limited. Meanwhile, there is currently no standardized criterion to assess the level of DS knowledge reservoir among individuals. In the present study, we designed 10 questions of varying difficulty to reflect the level of awareness and understanding of dietary supplements among individuals. Although the present study population is relatively young (nearly 60% of them were born in the 1990s and thereafter), less than 30% of them were able to correctly answer eight or more questions. Among all sociodemographic characteristic groups, only participants in the highest education level group showed relatively higher accuracy rates. No significant differences in knowledge level were observed between participants with bachelor’s degrees and those with other educational backgrounds, or between medical- and non-medical-related professionals. In a multi-centered cross-sectional study in Chinese universities, 58.9% of university students (61.5% medical students versus 56.4% non-medical students) reported supplement use, and 10.5% of medical students versus 8.3% non-medical students considered the main effectiveness of supplements is to treat diseases (*p* = 0.034). However, it is somehow reassuring to find that medical students have greater odds of correct answers toward knowledge on nutritional supplements (25). Another nationwide cross-sectional anonymous questionnaire survey of 30,635 participants reported that among the surveyed residents aged 18 to 55 years, only 23.0% were able to correctly identify the main purpose of nutrient supplementation as compensating for diet insufficient. This proportion was even lower among residents aged 55 years and above (34).

Generally, there are several factors contributing to the low DS-related knowledge level of both our study participants and the general population. First, the rapid growth of the DS market in China over the past decade has outpaced the dissemination of accurate and reliable information (35). This has resulted in a lack of standardized guidelines and educational resources for consumers. Second, the absence of a unified standard to evaluate individuals’ knowledge reservoirs further exacerbates the situation. Moreover, the vast amount of information from various sources, such as television, radio, and the Internet, along with asymmetric information about products of overseas origin, makes it challenging for consumers to access and understand relevant knowledge and information. Addressing these challenges and promoting education and awareness initiatives are crucial steps toward improving the supplement-related knowledge level of our participants.

According to the HAPA and AIDA behavior models and other health behavior models, individuals’ knowledge about supplements could be affected by their sociodemographic characteristics, such as educational background, income, and professions, and is crucial for building correct awareness, understanding, and promoting informed decision-making on healthy life choices such as DS use (36–38). However, within our study participants, a higher knowledge level does not guarantee accurate awareness of supplement use. No significant difference was found among individuals with varying knowledge levels in viewing treating specific medical conditions as their primary motivation for using DS. Furthermore, a higher knowledge level and awareness were not necessarily associated with DS purchases, even after accounting for crucial influencing factors such as diet quality and self-efficacy. This indicates a significant gap between knowledge, intention, and practice regarding DS use and could potentially lead to several unwise health decisions. Based on previous population-based studies, it is a common phenomenon that there is a gap between knowledge levels and health-related cognitions and behaviors (21). In Japan, where supplements are popular, and where there is a Health Food Network consumer navigation site to provide all official information about supplements freely for the public, people were found to have extremely low awareness of appropriate use, and the awareness of such information source was below 5% (19, 20). Even with guidance and advice from health professionals, people used supplements and medicines concomitantly, with an inappropriate perception of their safety and efficacy (21). In the United States, 15.1% of supplement users did not tell their physicians about using DS, and the proportion almost doubled during the past decade (39). To bridge the gap between supplement-related knowledge, awareness, and appropriate practice, it is quite necessary to promote evidence-based education, enhance regulatory oversight, and encourage critical thinking among consumers. Education campaigns, public knowledge platforms, and targeted training programs for healthcare workers, as well as for the general population, may help enhance the accessibility and dissemination of accurate nutrition-related knowledge. Currently, there are few studies and surveys specifically focused on Chinese people’s knowledge, intention, and practice of DS, resulting in a lack of comprehensive data in this area. More high-quality surveys based on behavioral theoretical frameworks are needed to further understand the needs of Chinese residents for DS in the new era. Such surveys will also help guide people to improve their knowledge and make informed health choices.

Our current study incorporates two behavioral theoretical models from the outset, allowing for a detailed analysis of the behavioral patterns related to healthcare products, from knowledge and perception to actual behavior. There are also several limitations about the present study. First, although we tried to ensure a balanced sample collection from the southern, central, and northern regions, it was still insufficient to explore the differences between regions. Due to the significant variations in dietary cultures across different regions in China, this could potentially lead to different perceptions and decision-making regarding health supplements. Second, there is currently no validated questionnaire specifically for assessing knowledge and awareness levels regarding health supplements. The questions used in this study regarding knowledge and cognitive behaviors related to health supplements were derived from experts’ experiences in previous research and consumer behavior models. Although they are similar to questions used in other studies, to better understand the relevant knowledge of the population, it is recommended to develop appropriate questionnaires based on more representative samples. Third, all of our data were collected from online self-reported questionnaires. Although we set strict restrictions on the logic values of each item, potential response quality issues may still affect the representativeness and reliability of the data. In addition, concerns about privacy and cultural differences may influence respondents’ engagement and interpretation of questions.

## Conclusion

There is a significant and growing demand for supplements among Chinese adults, with a trend toward younger demographics. However, the overall knowledge level of dietary supplements is relatively low, creating a considerable gap between knowledge, intention, and decision-making behaviors. To enhance public understanding and improve the ability to discern authentic information, it is essential to compile objective and neutral information on dietary supplements from various aspects and provide assessable knowledge sources to both healthcare professionals and the general population. Health education strategies should focus not only on improving health literacy but also on actively promoting the translation of knowledge into informed health decision-making.

## Data Availability

The raw data supporting the conclusions of this article will be made available by the authors, without undue reservation.
