# Supplementary material for: The association between the knowledge, perception, and practice of dietary supplement among Chinese adults
Source: Front Nutr. 2024 Nov 8;11:1493504. doi: 10.3389/fnut.2024.1493504 (PMC11583637; doi:10.3389/fnut.2024.1493504)
Supplement: Supplementary file 1 [file Data_Sheet_1.DOCX]

**Supplementary Figure 1.** Hypothetical framework on dietary supplement related behavior, based on HAPA (Health Action Process Approach) and AIDA (Attention, Interest, Desire, Action) models) models

**Supplementary Figure 2** Distribution of participants regarding the number of correctly answered questions on dietary supplements related knowledge.

**Supplementary Figure 3** Association between supplement related intention and supplement purchase behavior during the past 6 months, and average expenses on supplement.

Note: A. Crude model; B. Fully adjusted model. Expense on supplement were only calculated in participants who bought supplement during the past 6 months (N=924).

Fully adjusted model included for birth date, education background, occupation, yearly income, family members, exercise habit, self-efficacy (low or high), Dietary Diversity Score for the past 24 hours, and supplement related knowledge level.

| Supplementary Table 1 Questions to evaluate dietary supplement related knowledge | |
| --- | --- |
| Questions | Value assignment |
| 1.The key to health in dietary supplement lies in their ingredients and formulations. | No=0, Yes=1 |
| 2. Dietary supplement can replace medication. | Yes=0, No=1 |
| 3. Dietary supplement can treat diseases. | Yes=0, No=1 |
| 4. Dietary supplement are the best way to improve suboptimal health. | Yes=0, No=1 |
| 5. Dietary supplements, health supplements, and health foods are the same. | Yes=0, No=1 |
| 6. A blue cap signifies a dietary supplement in China. | No=0, Yes=1 |
| 7. If there is a calcium deficiency, calcium or related dietary supplements should be taken. | No=0, Yes=1 |
| 8. Regardless of the type, the supplementation of probiotics is beneficial for gut microbiota health. | No=0, Yes=1 |
| 9. Vitamin C is directly related to antioxidant effects but should not be supplemented long-term. | No=0, Yes=1 |
| 10. Clinical trials are required for dietary supplements. | No=0, Yes=1 |

One score was assigned for each correct-answered question. The total score ranged between 0 an 10, while higher scores indicated to better dietary supplement related knowledge.

| Supplementary Table 2 Association between sociodemographic characteristics, knowledge, and motivations for dietary supplement use | | | | | |
| --- | --- | --- | --- | --- | --- |
| Socio and demographic characteristics | Improving health | Medical conditions | Overall well-being | Beauty/ Cosmetics | Dietary deficiencies |
| Birth Date |  |  |  |  |  |
| 1960s | 1 (Ref.) | 1 (Ref.) | 1 (Ref.) | 1 (Ref.) | 1 (Ref.) |
| 1970s | 1.0 (0.6, 1.7) | 1.2 (0.6, 2.4) | 0.8 (0.4, 1.5) | 0.6 (0.3, 1.0) | 1.6 (0.9, 2.5) |
| 1980s | 0.9 (0.6, 1.5) | 1.1 (0.5, 2.1) | 0.8 (0.5, 1.5) | 0.8 (0.5, 1.4) | 1.5 (0.9, 2.3) |
| 1990s | 1.0 (0.6, 1.8) | 1.4 (0.7, 2.6) | 0.9 (0.5, 1.5) | 0.9 (0.6, 1.5) | 1.1 (0.7, 1.7) |
| 2000s | 0.7 (0.5, 1.2) | 1.0 (0.5, 2.0) | 1.1 (0.6, 1.9) | 1.0 (0.6, 1.6) | 1.3(0.8, 2.0) |
| Education Background |  |  |  |  |  |
| High school or below | 1 (Ref.) | 1 (Ref.) | 1 (Ref.) | 1 (Ref.) | 1 (Ref.) |
| Associate degree | 0.6 (0.4, 0.9) | 1.7 (0.9, 3) | 1 (0.6, 1.7) | 1 (0.7, 1.6) | 1.2 (0.8, 1.7) |
| Bachelor's degree | 0.6 (0.4, 0.9) | 1.5 (0.8, 2.6) | 0.9 (0.6, 1.5) | 1.1 (0.7, 1.6) | 1.3 (0.9, 1.8) |
| Master's degree or above | 1.1 (0.6, 1.8) | 1 (0.4, 2.4) | 1.1 (0.6, 2.2) | 0.6 (0.3, 1.1) | 1 (0.6, 1.7) |
| Occupation |  |  |  |  |  |
| Non-medical related professions | 1 (Ref.) | 1 (Ref.) | 1 (Ref.) | 1 (Ref.) | 1 (Ref.) |
| Medical marketing related | 0.9 (0.7, 1.2) | 1.1 (0.8, 1.7) | 1 (0.7, 1.5) | 0.9 (0.7, 1.2) | 1.1 (0.8, 1.4) |
| Medical research/teaching | 1 (0.7, 1.5) | 0.8 (0.5, 1.4) | 1 (0.6, 1.6) | 0.9 (0.6, 1.3) | 1.1 (0.8, 1.5) |
| Yearly income |  |  |  |  |  |
| <100k | 1 (Ref.) | 1 (Ref.) | 1 (Ref.) | 1 (Ref.) | 1 (Ref.) |
| 100-200k | 1.1 (0.8, 1.4) | 1.1 (0.7, 1.7) | 0.7 (0.5, 1.1) | 0.9 (0.7, 1.3) | 1.2 (0.9, 1.5) |
| >200-300k | 0.7 (0.5, 1.1) | 1.5 (0.9, 2.5) | 1 (0.6, 1.5) | 1 (0.6, 1.5) | 1.1 (0.8, 1.6) |
| >300-400k | 0.8 (0.4, 1.3) | 0.7 (0.3, 1.6) | 1.3 (0.7, 2.2) | 1.2 (0.7, 1.9) | 1.1 (0.7, 1.7) |
| >400-500k | 1.6 (1, 2.6) | 1.2 (0.6, 2.4) | 0.8 (0.4, 1.5) | 0.5 (0.3, 1) | 1 (0.6, 1.6) |
| >500k | 0.8 (0.5, 1.1) | 1.7 (1.1, 2.5) | 0.8 (0.5, 1.2) | 1 (0.7, 1.4) | 1.1 (0.8, 1.4) |
| Family members |  |  |  |  |  |
| 1 | 1 (Ref.) | 1 (Ref.) | 1 (Ref.) | 1 (Ref.) | 1 (Ref.) |
| 2~3 | 1.2 (0.9, 1.8) | 1.5 (0.9, 2.4) | 0.8 (0.5, 1.2) | 1 (0.7, 1.4) | 0.9 (0.7, 1.2) |
| 4~5 | 1 (0.7, 1.6) | 1.5 (0.9, 2.7) | 0.7 (0.5, 1.2) | 0.9 (0.6, 1.4) | 1.1 (0.8, 1.5) |
| ≥5 | 1.4 (0.9, 2.3) | 1.7 (0.9, 3.3) | 0.6 (0.4, 1.1) | 1.1 (0.7, 1.8) | 0.7 (0.5, 1.1) |
| Exercise Habit |  |  |  |  |  |
| No | 1 (Ref.) | 1 (Ref.) | 1 (Ref.) | 1 (Ref.) | 1 (Ref.) |
| Occasionally | 0.8 (0.6, 1.1) | 1.2 (0.8, 1.8) | 1 (0.7, 1.5) | 1.2 (0.9, 1.7) | 0.9 (0.7, 1.2) |
| Yes | 0.7 (0.5, 0.9) | 1 (0.7, 1.5) | 1.1 (0.8, 1.5) | 1.2 (0.9, 1.6) | 1.2 (0.9, 1.5) |
| Supplement knowledge level |  |  |  |  |  |
| Low | 1 (Ref.) | 1 (Ref.) | 1 (Ref.) | 1 (Ref.) | 1 (Ref.) |
| Middle | 1.1 (0.8, 1.5) | 1.6 (1, 2.5) | 1.1 (0.7, 1.5) | 0.8 (0.6, 1.1) | 0.8 (0.6, 1.1) |
| High | 1 (0.7, 1.5) | 1.5 (0.9, 2.4) | 1 (0.6, 1.4) | 0.9 (0.6, 1.2) | 0.9 (0.7, 1.2) |

| Supplementary Table 3 Association between sociodemographic characteristics, knowledge, and primary choice when there is discomfort symptoms. | | | | |
| --- | --- | --- | --- | --- |
| Socio and demographic characteristics | Supplements based on symptoms | Medication based on symptoms | Seeking medical advice online/offline | Ignore |
| Birth Date |  |  |  |  |
| 1960s | 1 (Ref.) | 1 (Ref.) | 1 (Ref.) | 1 (Ref.) |
| 1970s | 0.9 (0.5, 1.4) | 1.4 (0.8, 2.3) | 1.1 (0.7, 1.7) | 0.6 (0.3, 1.2) |
| 1980s | 1.6 (1, 2.5) | 1 (0.6, 1.7) | 0.7 (0.4, 1.1) | 0.9 (0.5, 1.6) |
| 1990s | 1.1 (0.7, 1.7) | 1.1 (0.7, 1.8) | 0.9 (0.6, 1.3) | 1 (0.6, 1.6) |
| 2000s | 1 (0.7, 1.6) | 1.5 (1, 2.4) | 0.8 (0.5, 1.1) | 0.8 (0.5, 1.4) |
| Education Background |  |  |  |  |
| High school or below | 1 (Ref.) | 1 (Ref.) | 1 (Ref.) | 1 (Ref.) |
| Associate degree | 1 (0.7, 1.5) | 1 (0.7, 1.5) | 0.9 (0.6, 1.3) | 1.2 (0.7, 2) |
| Bachelor's degree | 0.9 (0.7, 1.4) | 1 (0.7, 1.4) | 0.9 (0.7, 1.3) | 1.2 (0.8, 2) |
| Master's degree | 1.1 (0.6, 1.8) | 0.8 (0.5, 1.4) | 1.1 (0.6, 1.7) | 1.1 (0.5, 2.1) |
| Occupation |  |  |  |  |
| Non-medical related professions | 1 (Ref.) | 1 (Ref.) | 1 (Ref.) | 1 (Ref.) |
| Medical marketing related | 0.9 (0.7, 1.2) | 1 (0.7, 1.3) | 0.9 (0.7, 1.2) | 1.5 (1.1, 2.1) |
| Medical research/teaching related | 0.8 (0.6, 1.2) | 0.9 (0.6, 1.3) | 0.9 (0.6, 1.3) | 1.7 (1.2, 2.6) |
| Yearly income |  |  |  |  |
| <100k | 1 (Ref.) | 1 (Ref.) | 1 (Ref.) | 1 (Ref.) |
| 100-200k | 1.2 (0.9, 1.7) | 0.7 (0.5, 0.9) | 1.1 (0.9, 1.5) | 1 (0.7, 1.5) |
| >200-300k | 1.2 (0.8, 1.8) | 0.7 (0.4, 1) | 1.2 (0.8, 1.7) | 1 (0.6, 1.7) |
| >300-400k | 1 (0.6, 1.6) | 1 (0.6, 1.6) | 1 (0.7, 1.6) | 0.9 (0.5, 1.7) |
| >400-500k | 0.8 (0.5, 1.3) | 1.3 (0.8, 2.1) | 0.8 (0.5, 1.3) | 1.2 (0.7, 2.2) |
| >500k | 1.2 (0.9, 1.6) | 0.7 (0.5, 1) | 0.8 (0.6, 1.1) | 1.6 (1.2, 2.3) |
| Family members |  |  |  |  |
| 1 | 1 (Ref.) | 1 (Ref.) | 1 (Ref.) | 1 (Ref.) |
| 2~3 | 0.9 (0.6, 1.2) | 1 (0.7, 1.3) | 1 (0.8, 1.4) | 1.2 (0.8, 1.9) |
| 4~5 | 0.8 (0.6, 1.2) | 1 (0.7, 1.4) | 1 (0.7, 1.4) | 1.5 (0.9, 2.4) |
| ≥5 | 0.9 (0.6, 1.4) | 1.1 (0.7, 1.7) | 0.8 (0.5, 1.2) | 1.4 (0.8, 2.4) |
| 0 |  |  |  |  |
| Exercise Habit |  |  |  |  |
| No | 1 (Ref.) | 1 (Ref.) | 1 (Ref.) | 1 (Ref.) |
| Occasionally | 0.8 (0.6, 1) | 1.3 (1, 1.7) | 1 (0.8, 1.3) | 1 (0.7, 1.4) |
| Yes | 0.9 (0.7, 1.2) | 1.4 (1, 1.8) | 0.9 (0.7, 1.1) | 0.9 (0.7, 1.3) |
| Supplement knowledge level |  |  |  |  |
| Low | 1 (Ref.) | 1 (Ref.) | 1 (Ref.) | 1 (Ref.) |
| Middle | 1.1 (0.8, 1.4) | 0.7 (0.5, 0.9) | 1.4 (1.1, 1.8) | 0.9 (0.6, 1.3) |
| High | 0.7 (0.5, 1) | 1 (0.7, 1.3) | 1.6 (1.2, 2.2) | 0.8 (0.5, 1.1) |
